# Insecticidal Activity of *Bacillus thuringiensis* Strains Isolated from Soil and Water

**DOI:** 10.1100/2012/710501

**Published:** 2012-05-01

**Authors:** Edyta Konecka, Jakub Baranek, Anita Hrycak, Adam Kaznowski

**Affiliations:** Department of Microbiology, Faculty of Biology, Adam Mickiewicz University, Umultowska 89, 61-614 Poznań, Poland

## Abstract

We attempted to search novel *Bacillus thuringiensis* strains that produce crystals with potential utility in plant protection and with higher activity than strains already used in biopesticide production. Seven *B. thuringiensis* soil and water isolates were used in the research. We predicted the toxicity of their crystals by *cry* gene identification employing PCR method. The isolate MPU B63 with interesting, according to us, genes content was used in evaluating its crystal toxicity against *Cydia pomonella* caterpillars. The strain MPU B63 was cultured from water sample and had *cry1Ab*, *cry1B*, and *cry15* genes. The LC_50_ crystals of MPU B63 were compared to LC_50_ of commercial bioinsecticide Foray determined against *C. pomonella* (codling moth). The activity of MPU B63 inclusions against codling moth larvae was approximately 24-fold higher than that of Foray. The results are a promising introduction for further study evaluating the potential usefulness of isolate MPU B63 crystals in plant protection.

## 1. Introduction

Biopreparations based on spore-crystals mixtures of *Bacillus thuringiensis* seem to be a good alternative for chemical pesticides. They are environment friendly, do not have a negative influence on nontarget animals, including vertebrates, and are effective in reducing the number of insect pests [1]. However, novel *B. thuringiensis* isolates with higher and broader spectrum of activity are searching in their natural habitats. New strains are cultured from samples collected from soil [2], leaves [3], dead insects [4], and other sources [5–7].

 Bacteria *B. thuringiensis* produce crystals comprised of Cry and Cyt proteins active against insect pest [8]. Sixty-eight groups of Cry and 3 groups of Cyt toxins have been known [9]. The toxicity of the most Cry and Cyt proteins are determined [10]. The knowledge on crystal composition leads to prediction of its potential activity [5]. An effective tool in estimating the utility of crystals against pests is identification of genes coding for insecticidal toxins [11]. For example, genes *cry1*, *cry2*, *cry7*, *cry8*, *cry9*, *cry15*, *cry22, cry51*, and *cyt1* code for proteins active against *Lepidoptera* pests [10]. Similarly, *cry54* codes for protein that is harmful to moths [12]. Other *cry* genes determine the synthesis proteins toxic for insects of *Diptera* [13, 14], *Coleoptera*, *Hemiptera, Hymenoptera*, *Hemiptera, Siphonoptera* [10], *Homoptera, Orthoptera, *and *Phthiraptera *orders [15]. Furthermore, detection of *cry* genes by PCR method enables discovering genes of novel crystalline toxins [11].

The protection of plants against some insects can be difficult. An example is protection of fruit trees against codling moth (*Cydia pomonella*) from *Lepidoptera* order. The pest forms tunnels inside the fruit and is hardly available for insecticides dispersed on the fruit surface. Moreover, *C. pomonella* is resistant to most chemical pesticides [16].

 We cultured *B. thuringiensis* strains from samples of soil and water in searching for novel isolates synthesizing crystals with high and wide insecticidal activity. We determined the potential toxicity of their crystalline inclusions by detection of *cry* gene profiles with PCR technique. The isolate with interesting, according to us, gene content was used in evaluating its crystal activity against *C. pomonella* caterpillars.

## 2. Materials and Methods

### 2.1. Bacteria

Seven *Bacillus thuringiensis* strains were used in the study (Table 1). Six bacterial isolates were cultured from soil samples. One strain was obtained from water of forest stream. The soil samples of 1 g were suspended in 10 mL 0.85% NaCl and heated with shaking at 80°C for 12 min. Aliquots of 100 *μ*L of suspension were plated on *Bacillus cereus* selective agar with egg's yolk polimixin emulsion (Biocorp, Poland) [17]. Bacterial colonies were displaced onto M.B.Th medium [18]. After 5 days of incubation, the culture stained with amino black and Ziehl's carbol fuchsin [19] was examined applying a standard light microscope. *B. thuringiensis* strain was recognized when black crystals dyed black were noticed.

### 2.2. Prediction of *B. thuringiensis* Insecticidal Toxicity by Crystalline Protein Genes Detection

Identification of *cry* genes was conducted by using PCR method with specific primers. DNA of the strains was extracted by boiling the bacterial cells [20]. One *μ*g of bacterial DNA was added to the PCR mixture containing 2.5 *μ*L 10 × PCR buffer, 2.5 mM MgCl_2_, 1 *μ*L 5 mM dNTP, 0.25 *μ*M of appropriate pair of primers, 1 U of HiFi *Taq* DNA polymerase, and sterile distilated water to 25 *μ*L of suspension. The PCR reagents were purchased from Novazym (Poland) and Oligo.pl (Poland). The sequences of primers and temperature of PCR annealing for *cry1* gene were done according to Ben-Dov et al. [21]. The subgroups of *cry1* genes were identified according to Juárez-Pérez et al. [22], Monnerat et al. [23], and Masson et al. [24]. Masson et al. [24] also described the primers and PCR conditions for *cry6*. Primer sequences and PCR steps for *cry2* gene, its subgroups, and* cry3*, *cry4*, and *cry7*/8 genes were presented by Ben-Dov et al. [21]. PCR for the presence of *cry5*, *cry12*, *cry14*, *cry21*, *cry13*, *cyt1Aa* and *cyt1Ab* genes was conducted according to Bravo et al. [25]. Primer sequences and steps of PCR for *cry9* and the gene subgroups detection were accomplished as proposed by Ben-Dov et al. [26]. Identification of* cry15, cry16, cry18, cry20, cry22, cry25, cry26, cry28,* and *cyt2* genes was described by Ejiofor and Johnson [6]. The amplification for *cry10, cry17*, *cry24, cry27, cry29, cry30, cry32* and *cry40* genes was conducted as depicted by Ibarra et al. [13]. Identification of *cry19* and *cry39* genes was done according to instruction of Salehi Jouzani et al. [27].

The gene amplifications were carried out in MyCycler Termal Cycler (Bio-Rad, USA). The PCR products were electrophoresed in 1.5% agarose gel NOVA Mini (Novazym, Poland), stained with ethidium bromide and documented with Bio-Print V.99 System (Vilber Lourmat, France). The sizes of amplicons were estimated by GelCompar II 3.5 software (Applied Maths, Belgium).

### 2.3. Activity of *B. thuringiensis* Crystals against *C. pomonella* Caterpillars

The activity of *B. thuringiensis* crystals against *C. pomonella* was determined with applying the strain MPU B63 with *cry1Ab*, *cry1B,* and *cry15* genes. The *B. thuringiensis* strain was cultured on M.B.Th medium for 5 days during sporulation. The mixture of spores and crystals was collected, washed with 1 M NaCl and then in distilated sterile water [28]. The spore-crystal mixture was suspended in 50 mM Tris HCl, 10 mM KCl, and pH 7.5 and placed on sucrose density gradient (67%, 72%, 79%, and 87%). After centrifugation, the layer of crystals was gathered and washed in sterile distilated water [29].

The number of crystals in the suspension was evaluated in a Bűrker cell. Five dilutions of toxins (10^2^–10^6^) were prepared and applied to two-day-old *Cydia pomonella* caterpillars. The spore-crystal mixture of commercial pesticide Foray was prepared in the same manner, at the same time, and using the same conditions as for MPU B63. The larvae were cultured on medium according to Guennelon et al. [30]. The suspension of MPU B63 crystals or Foray spore-crystal preparation with known concentrations was spread on the medium surface. The larvae are cannibalistic; therefore, they were reared individually at a 16 : 8 (day : night) period, 26°C and 40–60% humidity. The number of dead insects was estimated after 7 days.

The 50% lethal concentration (LC_50_) of MPU B63 crystals against *C. pomonella* was calculated by using probit analysis with the consideration of dead caterpillars in control sample [31]. The obtained value was compared to LC_50_ commercial bioinsecticide Foray determined against *C. pomonella*. The insecticidal activity of Foray preparation is 21200 IU/mg. The potency (IU/mg) of isolate MPU B63 was counted using the following formula [32]: potency of isolate crystals (IU/mg) = [LC_50_ of Foray × potency of Foray (IU/mg)]/LC_50_ of isolate crystals.

## 3. Results

### 3.1. Distribution of Crystalline Toxin Genes

The *B. thuringiensis* strains had from three to eight crystalline toxin genes. We found that the isolates had *cry1Aa, cry1Ab, cry1Ac, cry1B*,* cry1C*,* cry1D, cry1I, cry2Aa cry2Ab, cry9B*,* cry9E,* and *cry15.* The obtained results are given in Table 1.

**Figure 1:**
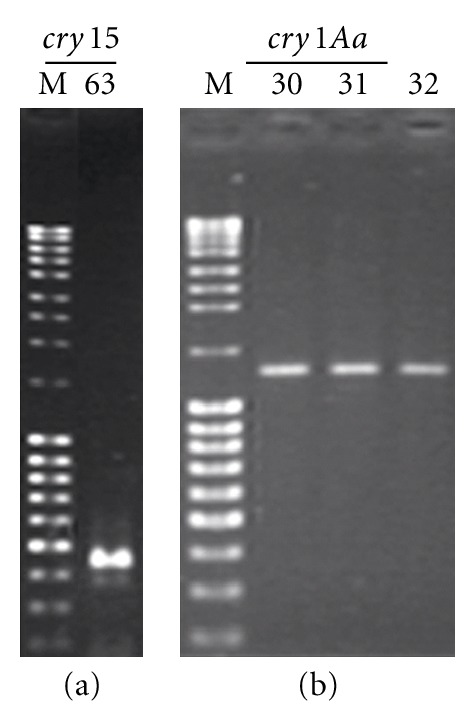
Amplicon of *cry15* gene of *B. thuringiensis* MPU B63 strain is presented in panel a on the left. Amplicons of *cry1Aa* gene of *B. thuringiensis* MPU B30, MPU B31, and MPU B32 strains are presented in panel b on the right. Lanes: M. MassRuler DNA Ladder, Mix (10000, 8000, 6000, 5000, 4000, 3000, 2500, 1500, 1031, 900, 800, 700, 600, 500, 400, 300, 200 bp), 63. *B. thuringiensis* MPU B63, 30. *B. thuringiensis* MPU B30, 31. *B. thuringiensis* MPU B31, 32. *B. thuringiensis* MPU B32.

*B. thuringienis* soil isolates harbored *cry1*, *cry2* and *cry9* genes. Strain MPU B63 cultured from water possessed *cry1* and *cry15* genes. The *cry1A* gene was present in all isolates. All *B. thuringiensis* strains obtained from soil samples carried *cry2A* and *cry1I*. Strains with *cry1C* had also *cry1D*, *cry9B,* and *cry9E*. The soil isolate MPU B30 had large number and diversity of *cry* genes; it possessed *cry1Aa*,* cry1B*,* cry1C*,* cry1D*,* cry1I*,* cry2Ab*,* cry9B*, and* cry9E* genes. The amplicons of some* cry* gene are shown in Figure 1. None of the isolates had *cry1J, cry1K*, *cry5, cry6, cry7, cry8, cry11, cry12, cry13, cry14, cry16, cry17*, *cry18, cry19, cry20, cry21, cry22, cry24, cry26, cry27, cry28, cry29, cry30, cry32, cry 39, cry40, cyt1,* and *cyt2* genes. 

### 3.2. Toxicity of *B. thuringiensis* MPU B63 Crystals for *Cydia pomonella* Larvae

The strain MPU B63 was chosen to determine its crystal activity due to unique *cry* gene profile. The isolate had *cry15* gene. The LC_50_ value of MPU B63 toxins against *C. pomonella* was 1.55 × 10^5^ crystals per larva. The obtained value was compared to the LC_50_ of commercial biopesticide Foray containing spores and crystals of *B. thuringiensis* subsp. *kurstaki* that is recommended to protect plants against lepidopteran insects. LC_50_ of Foray for *C. pomonella* was 3.69 × 10^6^ spores and crystals per larva (Table 2). The LC_50_ of MPU B63 crystals was approximately 24-fold lower than LC_50_ of bioinsecticide against *C. pomonella* caterpillars. The potency of MPU B63 toxins was approximately 890 IU/mg, and it was higher than the potency of Foray.

## 4. Discussion

*Bacillus thuringiensis* bacteria are ubiquitous in soil [2, 13, 33, 34], dead larvae [4], sand [5], leaves [3], water [7], or dust from stored grains [6]. Wild strains isolated form environmental samples can synthesize crystals that display higher activity against insect pests in comparison to *B. thuringiensis* strains already used in pesticide production. We attempted to culture *B. thuringiensis* isolates from soil and water samples and estimate their potential usefulness in plant protection.

 The knowledge on coding for genes toxins in crystalline inclusion is useful in predicting potential pathogenicity of *B. thuringiensis* isolates against insects [5, 7, 11]. Cry1 toxins display activity against lepidopteran, dipteran, and coleopteran pests. *Cry2* genes code for crystalline proteins toxic for *Diptera* and *Hemiptera*. Cry9 proteins indicate activity against insects of *Coleoptera* and *Lepidoptera* order. Cry15 is toxic for lepidopteran pests [10]. Two of soil-isolated strains (MPU B30 and MPU B55) had genes of Cry1, Cry2, and Cry9 toxins. Other *B. thuringiensis* isolates cultured from soil possessed *cry1* and *cry2* genes. Their crystals showed potential activity against pests of *Coleoptera*, *Diptera*, *Hemiptera,* and *Lepidoptera*. Water-isolated strain harbored genes coding Cry1 and Cry15 toxins that indicate the crystals activity against coleopteran, dipteran, and lepidopteran insects.

All isolates had *cry1* gene, and seven of eight strains harbored *cry2* gene. These genes were also noted as the most frequent in *B. thuringiensis* strains [2, 3, 5, 6, 33, 34]. All analyzed *B. thuringiensis* harbored *cry1I* genes that have been reported as the most abundant in *B. thuringiensis* isolates [11]. Soil-isolated strains with *cry1A* possessed also *cry2A* gene, which is with agreement in notice done by Saadaoui et al. [3] in strains from soil samples collected in Tunisia. We observed that strains with *cry1C* had also *cry1D*, *cry9B,* and *cry9E*.

Strain *B. thuringiensis* subsp. *kurstaki* HD-1 applied in production of insecticide Foray harbored *cry1Aa, 1Ab, 1Ac, 1I, 2Aa, 2Ab, *and* 2Ac* genes [35]. Soil isolate MPU B30 had the largest number of *cry* genes among the isolates analyzed (Table 1). In comparison to Foray, it additionally carries *cry1B, cry1C, cry1D, cry9B,* and *cry9E* genes, which can indicate wider spectrum of toxicity and higher insecticidal activity of their crystals than the commercial insecticide. Our attention was directed to MPU B63 with *cry15* gene isolated from water sample. The gene is rarely detected in environmental isolates [6], and only a few reports about its activity against lepidopteran insects have been published [36–38]. We isolated crystalline inclusions of MPU B63 strain, evaluated their insecticidal activity towards *C. pomonella*, and compared with the activity of Foray recommended to protect plants against insect of *Lepidoptera* order.

 The activity of MPU B63 crystals against *C. pomonella* caterpillars was approximately 24-fold higher than Foray pesticide. It indicates the contribution of MPU B63 toxins that are not possessed by *B. thuringiensis* subsp. *kurstaki* HD-1 used in Foray production. Strain MPU B63 had *cry1B* and *cry15* genes that were not identified in HD-1 strain. The activity of Cry1B [39] and Cry15 [37] proteins for codling moth has been reported. Cry15 is a binary toxin. It occurs in crystal together with another protein of 40-kDa molecular mass that is active only with the presence of Cry15 [36, 40]. According to Naimov et al. [37], the role of 40-kDa protein is to form crystal and to achieve higher Cry15 levels. Its absence results in Cry15 degradation. The mode of action of binary crystalline toxins is unknown, but it was found that Cry15 has nonspecific pore-forming activity and displays hemolysis on mouse erythrocytes [37].

 Our searching for a novel isolate producing crystals with higher activity than commercial biopesticide revealed the MPU B63 strain. The toxicity of Foray insecticide was approximately 24-fold lower compared to that of MPU B63 crystals. The results are a starting point for future research determining potential usefulness of MPU B63 isolate in plant protection.

## Figures and Tables

**Table 1:** Genes of *Cry* toxins in *B. thuringiensis *isolates cultured from samples of soil and water.

Strain designation	Source of isolation or reference	*cry* genes
MPU B^1^30	Soil	*cry1Aa*,* 1B*,* 1C*,* 1D*,* 1I*,* 2Ab*,* 9B*,* 9E *
MPU B31	Soil	*cry1Aa*,* 1Ab*,* 1Ac*,* 1I*,* 2Aa*,* 2Ab *
MPU B32	Soil	*cry1Aa*,* 1Ab*,* 1Ac*,* 1I*,* 2Aa*,* 2Ab *
MPU B55	Soil	*cry1Aa*,* 1C*,* 1D*,* 1I*,* 2Ab*,* 9B*,* 9E *
MPU B61	Soil	*cry1Ab*,* 1Ac*,* 1I*,* 2Aa*,* 2Ab *
MPU B62	Soil	*cry1Ac*,* 1I*,* 2Aa*,* 2Ab *
MPU B63	Water	*cry1Ab*,* 1B*, *15 *

^1^ Collection of Department of Microbiology, Adam Mickiewicz University, Poznań, Poland.

**Table 2:** The 50% lethal concentration (LC_50_) of crystals of *B. thuringiensis *MPU B63 and Foray for *C. pomonella* caterpillars.

Preparation	LC_50_ value on insect caterpillars [crystals per larva]	Confidence interval 95%
Crystal mixture of MPU B63	1.55 × 10^5^	5.89 × 10^4^–4.11 × 10^5^
Spore-crystal mixture of Foray	3.69 × 10^6^	5.7 × 10^5^–2.39 × 10^7^
